# Opioid Use Disorder Curriculum: Preclerkship Pharmacology Case-Based Learning Session

**DOI:** 10.15766/mep_2374-8265.11255

**Published:** 2022-05-10

**Authors:** Sabrina Taldone, Sandra Lemmon, Suzy Bianco, Joan St. Onge, Henri Ford, Lindsay Cox, David P. Serota, Sabita Roy, Jason Onugha, David W. Forrest, Tyler Bartholomew, Hansel E. Tookes

**Affiliations:** 1 Assistant Professor, Department of Medicine, University of Miami Leonard M. Miller School of Medicine; 2 Professor, Department of Molecular and Cellular Pharmacology, University of Miami Leonard M. Miller School of Medicine; 3 Pharmacist, Department of Molecular and Cellular Pharmacology, University of Miami Leonard M. Miller School of Medicine; 4 Professor, Department of Medicine, University of Miami Leonard M. Miller School of Medicine; 5 Dean and Chief Academic Officer, University of Miami Leonard M. Miller School of Medicine; 6 Resident, Department of Psychiatry, University of Miami Leonard M. Miller School of Medicine; 7 Professor, Department of Surgery, University of Miami Leonard M. Miller School of Medicine; 8 Resident, Department of Medicine, University of Miami Leonard M. Miller School of Medicine; 9 Associate Professor, Department of Public Health Sciences, University of Miami Leonard M. Miller School of Medicine; 10 Assistant Professor, Department of Public Health Sciences, University of Miami Leonard M. Miller School of Medicine; 11 Associate Professor, Department of Medicine, University of Miami Leonard M. Miller School of Medicine

**Keywords:** Opioid Use Disorder, Case-Based Learning, Internal Medicine, Pharmacology & Toxicology, Opioids, Addiction, Pain

## Abstract

**Introduction:**

During the first year of the COVID-19 pandemic, over 93,000 Americans lost their lives to a preventable overdose. Medications for opioid use disorder (OUD) have been shown to decrease mortality in OUD but are underutilized. Through this case-based learning exercise, first-year medical students applied physiologic and pharmacologic principles to the diagnosis and treatment of OUD.

**Methods:**

Faculty facilitated a case discussion over a 1-hour large-group case-based learning (CBL) session. Facilitators utilized PowerPoint slides to illustrate graphs and figures while discussing the case. To evaluate students on the CBL learning objectives, three pharmacology exam questions were administered; students also evaluated the CBL's effectiveness in meeting educational objectives on three Likert-scale questions and via open-ended feedback.

**Results:**

First-year medical students (*n* = 200) completed the CBL. The mean score on the exam questions was 91%. Students agreed or strongly agreed that the CBL was an effective way to learn pharmacology principles (69%), that it reinforced pharmacologic fundamentals (70%), and that it showed how pharmacology fundamentals were important in the real world of clinical medicine (86%). Qualitative feedback on the CBL was generally positive, including satisfaction with the small-group setting and practical applications of pharmacology to clinical practice.

**Discussion:**

This CBL exercise contains content critical for preparing students to combat the modern opioid epidemic. The exercise provides an opportunity for learners to review fundamental pharmacodynamic and pharmacokinetic principles so as to ready them for clinical clerkships and beyond.

## Educational Objectives

By the end of this activity, learners will be able to:

1. Describe the physiologic effects and pharmacology of opioids.
2. Apply the pharmacology of opioids to the medications used to treat opioid use disorder.
3. Explain how pharmacology fundamentals are important in the real world of clinical medicine.

## Introduction

During the first year of the COVID-19 pandemic, over 93,000 Americans lost their lives to a preventable overdose, with 69,710 deaths due to opioid overdose, including powerful fentanyl and analogues.^1^ People living with untreated opioid use disorder (OUD) have over six times the mortality of the general population; however, with lifesaving medications for OUD (MOUD), the mortality for people living with OUD drops to less than two times the mortality of the general population.^2–4^ Unfortunately, these lifesaving medications are greatly underutilized in clinical practice.^5^ Through this case-based learning (CBL) exercise, first-year medical students applied physiologic and pharmacologic principles to the diagnosis and treatment of OUD, with the hope that the exercise would help to address the underutilization of MOUD in clinical practice through early exposure to these concepts among all medical students.

Perceived lack of comfort with MOUD extends beyond students.^6^ In 2021, only 37% of primary care physicians were somewhat or very comfortable in treating patients with OUD with medications.^7^ Therefore, we began with faculty development, hosting a Drug Addiction Treatment Act of 2000 (DATA) waiver training for 115 of our faculty and residents and certifying two of our faculty to be DATA waiver instructors.^8,9^ The DATA waiver enables office-based treatment of OUD.^9^ As of April 2021, with recent rule changes by the administration of President Joseph R. Biden allowing all physicians to prescribe MOUD to up to 30 patients without undergoing the 8-hour training required under DATA,^10^ medical schools have the timely opportunity to integrate pharmacology of MOUD into undergraduate medical education and overcome the lack of DATA waiver providers nationwide.^11^ Published literature on education for MOUD has been somewhat limited to faculty and residents in the fields of psychiatry and medicine.^12–14^ In a 2019 survey, only 15% of internal medicine residency programs reported “very effective” teaching of treatment of OUD.^15^ There is scant research on the comfort of entering PGY-1s (interns) with prescribing MOUD.

Given the expansive impact of people with OUD on all fields of medicine, it is imperative that medical students develop foundational knowledge of opioids during preclinical training before they encounter the clinical clerkships. In a scoping review of substance use disorder education in medical schools, only one study on OUD met inclusion criteria; the authors concluded that there was a need for increased training in OUD, including use of the CBL format.^16^ Some headway has been made by undergraduate medical educators. At Brown University, the curriculum prepares all medical students to obtain a DATA waiver upon graduation.^17^ Part of that curriculum includes an interprofessional education workshop for preclerkship students that has resulted in improvements in students' knowledge as evaluated by the Opioid Overdose Knowledge Scale; this scale primarily deals with identifying and treating opioid overdose.^18,19^ The published work from that clerkship curriculum did not evaluate medical student readiness to prescribe MOUD. Other preclerkship curricula in the literature focus on various areas surrounding OUD, from opioid use in pain management^20,21^ to opioid risk-mitigation strategies (e.g., using naloxone to treat opioid overdose).^22^ *MedEdPORTAL* even hosts a collection of publications on opioids, addiction, and pain medicine.^23^

To our knowledge, medical education research has not described preclerkship curricula demonstrating how the basic sciences, like pharmacology, can be applied to CBL in OUD. We identified an educational opportunity at the University of Miami Leonard M. Miller School of Medicine to introduce students to the pharmacology of MOUD early in their preclinical curriculum in order to best prepare them for their clinical practice across specialties. Preclerkship students generally respond better to the contextualization of pharmacology disciplines in clinical medicine—connecting theory to practice for a deeper level of learning.^24^

Introducing the pharmacology of MOUD is a unique opportunity to review fundamental concepts of pharmacodynamics such as full agonism (i.e., methadone), partial agonism (i.e., buprenorphine), antagonism (i.e., naltrexone), and receptor affinity.^25,26^ It also provides an opportunity to introduce pharmacokinetic principles such as bioavailability (e.g., of buprenorphine in buprenorphine-naloxone combination product) and metabolism. In the case presented here, we offer a clinical vignette of a real patient living with OUD^27^ and introduce MOUD. Furthermore, themes such as the principle of harm reduction (meeting people who use drugs where they are), respecting patient autonomy, and treating people with compassion could be presented.

## Methods

This CBL session was part of the first-year medical students' pharmacology course in winter 2019. The CBL was the first part of an OUD longitudinal curriculum extending from the first-year preclerkship courses through the third-year core clerkships. It was a mandatory session worth 2 points (out of 100) in the course. As part of the legacy curriculum, the course was taught at the end of the first semester after students had already completed anatomy; molecular biology; genetics; host defense, pathogens, and pathology; and cellular function and regulation. The pathophysiology curriculum of organ systems was taught later in the first year in different courses. Students were provided with only the instructions included in Appendix A to prepare them for a CBL (no other guidance was provided to help them adapt to a CBL learning style). In addition to the case presented here, there was one other CBL session on physiology principles (not related to OUD) that students completed after this case (within the same week). Earlier in the semester, these first-year students had already engaged in learning the foundations of social determinants of health and communications skills through small-group sessions, standardized patient encounters, and didactic sessions.

Students received a lecture on the foundations of pharmacokinetics and pharmacodynamics less than a week prior to this CBL. The content of that previous lecture reviewed core basics of preclinical pharmacology principles as required by the Liaison Committee on Medical Education. Session objectives and optional preparatory resources (see Appendix A) were distributed 1 week ahead of the in-class CBL activities. Students received the student version of the case (Appendix B) prior to the small-group sessions. Students were expected to have reviewed and prepared their own answers independently before the small-group session. Of note, this case was the first CBL activity to which the medical students were exposed. In our medical school, CBL was scheduled as needed based on the course; for example, later on in the second semester, students participated in CBLs during the cardiology course.

The pharmacology course instructor assigned students into small groups of 10–15 people. Two faculty facilitators—one physician and one pharmacology faculty—were assigned to each small group in order to provide expertise on clinical and pharmacology-related aspects of the case and to demonstrate interprofessional communication. Clinical faculty were from multiple specialties, including general internal medicine, hospital medicine, infectious disease, psychiatry (general and addiction subspecialists), and emergency medicine. Small-group team size was determined based on faculty availability. Lead faculty (Sabrina Taldone and Hansel E. Tookes) facilitated a preparation session on the case for the faculty facilitators prior to the student sessions.

Faculty facilitated the case discussion using the facilitator guide, which included timing instructions to complete the case within 1 hour (Appendix C). Each small group was conducted in a room with a computer and a large television screen that displayed the PowerPoint from the computer. Facilitators utilized PowerPoint slides (Appendix D) to illustrate graphs and figures while discussing the case. The slides were identified in the facilitator guide, which also contained a facilitator script (Appendix C). Faculty were instructed to provide all answers shown in the facilitator guide during the session.

To evaluate students on the CBL learning objectives, we used a mixed-methods approach after completion of the CBL. Three test questions (Appendix E) were administered as part of the students' pharmacology examination, which took place 1 week after the CBL session. Two questions were part of the National Board of Medical Examiners (NBME) Customized Assessment in our cellular function and regulation course (exact questions not available for publication per NBME policy). These retired NBME questions were chosen based on their assessment of opioid pharmacology and are available to all medical schools. Students were asked to evaluate the CBL session as part of the pharmacology course evaluation, which included three questions specific to the CBL session (Appendix F). Responses to the questions were scored on a 4-point Likert scale (ranging from *Strongly Agree* to *Strongly Disagree*). In addition, students were asked to describe their satisfaction with the CBL session through open-ended questions. Descriptive statistics (percentages) of the students' responses to the three quantitative questions on the Likert scale were reported. Representative quotes from the open responses were reported based on themes identified through a structured coding frame (positive perceptions, negative attitudes).

## Results

Two hundred first-year medical students completed the CBL. The mean score on the three pharmacology exam questions (which were based on learning objectives from the CBL) was 91%. On NBME question 1, 96% of our students scored correctly, whereas 93% of Step 1 takers scored correctly. On NBME question 2, 84% of our students scored correctly, whereas 75% of Step 1 takers scored correctly. The third question was developed by one of the pharmacology authors of this activity (Sandra Lemmon), and 89% of our students scored correctly on this question (Appendix E).

The course evaluation results are presented in the Table. Based on student feedback, students agreed or strongly agreed that the CBL was an effective way to learn pharmacology principles (69%), that it reinforced pharmacologic fundamentals (70%), and that it showed how pharmacology fundamentals were important in the real world of clinical medicine (86%).

**Table.**
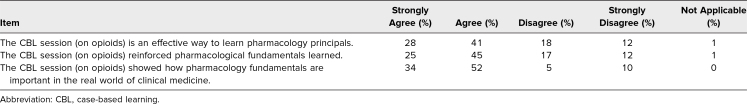
Results From Student Evaluations of the CBL Exercise (*n* = 82)

Students reacted positively to the CBL in their pharmacology course evaluation. They expressed satisfaction with the small-group setting and enjoyed the content covered during the section. Student comments included the following:

• “There was an instructor for our small group… who was the most insightful authoritative figure we were exposed to in the course. I enjoyably learned a lot from her.”
• “I thought the opioid section was fantastic!”

Students also felt that the CBL session on opioids had more practical applications to clinical practice compared to previous courses on pharmacology. One student said, “CBLs were educational and gave practical application of pharmacology. Highlighted the clinical aspects of medicine rather than the course being based on rote memorization.”

Not all students expressed support for the CBL. However, those students did provide constructive criticism on how to improve the design and delivery of the CBL in the future, including increasing transparency in the evaluation of students' performance. One student said, “I think that CBL questions are great, but with no actual answer key being released, there is no certainty that students have adequate understanding of the material.”

This case was the first time that these students had learned in a CBL format. This led to some feedback regarding perceptions of this different learning style, as compared to the usual lecture-based learning. Note that this session was part of a pass-fail preclerkship curriculum. One student said, “I think the time that went into preparing for the CBL/mandatory sessions and then participating in the sessions took a LOT of time out of our schedules to study for the exam.”

## Discussion

To address the dearth of preclerkship medical education curricula on MOUD and the pharmacologic principles underlying MOUD, we developed a CBL for first-year medical students. As an introduction to OUD, students worked together to frame the basics of opioid agonist, partial agonist, and antagonist pharmacology using drugs important in the opioid crisis: heroin, methadone, buprenorphine, naltrexone, and naloxone. The fundamental concepts in the pharmacology of opioids were presented through the lens of MOUD initiation, highlighting affinity for the mu opioid receptor and precipitated withdrawal, as well as overdose treatment. We detailed physiologic effects of opioids and withdrawal, introducing the Clinical Opioid Withdrawal Scale^28^ and tolerance to opioids. We introduced how the terminology used to describe people with substance use disorders affects stigma and health care quality.

It is clear from our results that the curriculum was both feasible and acceptable to students, with a high level of learner satisfaction. Eighty-six percent of students agreed or strongly agreed that the CBL showed how pharmacology fundamentals were important in the real world of clinical medicine. Our students performed above national average on two test questions from the NBME based on concepts from the CBL as well.

As faculty, we learned several important lessons from the design and implementation of this curriculum. We found the pharmacology of MOUD to be a particularly effective way to reinforce fundamental pharmacodynamic and pharmacokinetic principles that was acceptable to both faculty and students. However, the content was expansive for the time allotted—in future years, additional time for the small group would help facilitate deeper discussion of core concepts. Based on students' feedback on the real-world application of the case, we are hopeful that we have piqued their interest in MOUD, which could have lifesaving effects on patients they encounter in their clinical years of medical school, residency, and beyond. In hindsight, we should have given the students a broad overview of when the different aspects of the OUD longitudinal curriculum would be taught so that they would not feel desperate to address tangential topics during the CBL session. Also, we could have provided the students with a better expectation of what questions would be appropriate during CBL versus after class. A final lesson learned is that students could benefit from receiving a copy of the facilitator guide after the CBL to reference in preparation for examinations.

Limitations include the large number of faculty required to execute simultaneous small groups. Note that this limitation was overcome in subsequent years by using a smaller number of facilitators for repeated sequential sessions via remote videoconferencing during the COVID-19 pandemic. This CBL was presented in person to first-year medical students who were in the legacy curriculum model, which was primarily lecture based. The CBL was the first time that these students were exposed to a CBL format, with little instruction on how to prepare for it. Students were provided only with the instructions included in Appendix A to prepare them for a CBL (no other guidance was offered to help them adapt to a CBL learning style), so student perception of the benefit of CBL may have been subjective based on how implementation of CBL occurred in the curriculum. In the following year, fall 2020, the Miller School's curriculum was completely redesigned to be primarily based on CBL, team-based learning, and other flipped classroom models. Another limitation was in the assessment of knowledge acquisition: The CBL's expansive content was only able to be assessed in three test questions on the pharmacology exam (two NBME questions, one developed by the course director). Of note, NBME questions were chosen because of the emphasis on United State Medical Licensing Exam scores in residency application. We found it important to show our students that the OUD CBL was an innovative way to prepare them for these high-stakes exams. Also, the CBL was conducted at a single academic institution. It may not be generalizable to all medical schools, although we have included all the tools necessary to implement this exercise in the appendices and schools should have access to NBME Customized Assessments.

In order to curb the opioid epidemic across the US, it is urgent that medical schools prepare students to care for patients with OUD. The fundamentals of opioid pharmacology and physiology taught in this CBL will be enhanced during subsequent activities in the Miller School's longitudinal substance use disorder (OUD) curriculum, which reviews different aspects of opioid use and misuse, including neurobiology, clinical manifestations, pharmacology of opioids, pathophysiology of addiction and withdrawal, co-occurring substance use and mental health disorders, community resources, rehabilitation, and public health implications. The overarching principle of our curriculum centers upon cultural sensitivity when caring for patients with OUD. We have developed a longitudinal curriculum throughout the preclerkship and clerkship years that further expands on patient-centered care and persons with OUD; this case only briefly introduces concepts related to stigma affecting people with OUD via the description of the interactions between patient and nurse. Medical school curricula will need to incorporate more structured training on substance use disorder, which our curriculum at the Miller School aims to model and disseminate.

Overall, this essential CBL exercise contains content critical for preparing our students to combat the modern opioid overdose crisis. The longitudinal curriculum on OUD at our institution further addresses opioid withdrawal, overdose treatment, recovery, stigma, and even an OSCE to assess motivational interviewing, SBIRT (screening, brief intervention, and referral to treatment), harm reduction, and prescribing MOUD. This case introduced not only MOUD but also harm reduction and treating people with OUD with compassion. Finally, it provided an opportunity for learners to review fundamental pharmacodynamic and pharmacokinetic principles. This CBL was an effective modality to prepare our students for clinical clerkships and beyond.

## Appendices


Case Instructions and Resources.docxCase - Student Version.docxCase - Facilitator Guide.docxCase - Figures.pptPharmacology Exam Questions.docxEvaluation Questions.docx
*All appendices are peer reviewed as integral parts of the Original Publication.*
