# Supplementary material for: Opioid Use Disorder Curriculum: Preclerkship Pharmacology Case-Based Learning Session
Source: MedEdPORTAL. 2022 May 10;18:11255. doi: 10.15766/mep_2374-8265.11255 (PMC9085981; doi:10.15766/mep_2374-8265.11255)
Supplement: Supplementary file 1 — Case Instructions and Resources.docxCase - Student Version.docxCase - Facilitator Guide.docxCase - Figures.pptPharmacology Exam Questions.docxEvaluation Questions.docx [file mep_2374-8265.11255-s001.zip › A. Case Instructions and Resources.docx]

**Instructions for Students:**

**CBL**

**A. Objective:**

**Educational Objectives:**

By the end of this activity, learners will be able to:

• Describe the physiologic effects and pharmacology of opioids

• Apply the pharmacology of opioids to the medications used to treat opioid use disorder

• Explain how pharmacology fundamentals are important in the real world of clinical medicine

This CBL is based on an opioid clinical case. It was created by pharmacology faculty in collaboration with clinical faculty who have experience in treating patients with opioid use disorder. The goal of the session is to reinforce pharmacology fundamentals students already learned, and learn how these fundamentals apply in the real world of clinical medicine. You will already have had lectures on the following topics and tested on part of it:

Pharmacodynamics, Pharmacokinetics, Drug Metabolism, Pharmacogenetics/genomics

G coupled receptors, Autonomics

Some lectures in Physiology are relevant to this case.

To prepare for the session, we recommend that you review the material you have already learned in class AND do research on your own to answer the questions/topics in the CBL study guide provided. We expect you to come prepared for the in-class session to discuss the clinical case and underlying pharmacology. You will get the most out of the session if you prepare in advance. (See section D below for some useful resources)

**B. Room assignments:** There are 8 groups for this CBL exercise. There are two faculty leaders for each group, a basic science and a clinical faculty member.

**C. Other**

- You must attend the small group session to which you were assigned. Please be prompt.
- The faculty leaders will expect all students to participate. Please give your name if you are answering a question, so they can note it on their rosters.

**D. Optional resources:**

Recommended videos on opioids and opioid use disorder:

Khan Academy. Treatments and triggers for drug dependence. <https://youtu.be/tPhcRBkVmUM>

June 25, 2014. Accessed September 24, 2021.

Medicurio. Opioid Drugs, Part 1. Mechanism of Action. <https://youtu.be/s60KzN4GJdQ> June 28, 2018. Accessed September 24, 2021.

DRUGBANK

Wishart DS, Feunang YD, Guo AC, et al. DrugBank 5.0: a major update to the DrugBank database for 2018. *Nucleic Acids Res*. 2018;46(D1):D1074-D1082.

<https://academic.oup.com/nar/article/46/D1/D1074/4602867>

Link: <https://go.drugbank.com/>

PubChem

Kim S, Chen J, Cheng T, et al. PubChem in 2021: new data content and improved web interfaces. Nucleic Acids Res. 2021;49(D1):D1388–D1395. doi:10.1093/nar/gkaa971

Link: <https://pubchem.ncbi.nlm.nih.gov/>

Katzung BG, Vanderah TW. Katzung B.G., & Vanderah T.W.(Eds.),Eds. Bertram G. Katzung, and Todd W. Vanderah.eds. *Basic & Clinical Pharmacology, 15e*. McGraw Hill; 2021.
