# Supplementary material for: Opioid Use Disorder Curriculum: Preclerkship Pharmacology Case-Based Learning Session
Source: MedEdPORTAL. 2022 May 10;18:11255. doi: 10.15766/mep_2374-8265.11255 (PMC9085981; doi:10.15766/mep_2374-8265.11255)
Supplement: Supplementary file 1 — Case Instructions and Resources.docxCase - Student Version.docxCase - Facilitator Guide.docxCase - Figures.pptPharmacology Exam Questions.docxEvaluation Questions.docx [file mep_2374-8265.11255-s001.zip › B. Case - Student Version.docx]

**Medications for Opioid Use Disorder: Pharmacology Case-Based Learning Session**

**Section 1:**

Symptom: Upper extremity pain and swelling.

Mr. G is a 40-year-old man who presents to the Syringe Services Program with a chief complaint of a 3-day history of worsening right upper extremity pain and swelling. His medical history is significant for opioid use disorder, and he reports recent syringe sharing and re-use.

On physical exam, the patient is afebrile. The distal right upper extremity is swollen, warm, erythematous, and tender to light palpation. Point of care ultrasound shows an abscess on the ventromedial/flexor surface of his forearm. Rapid HIV and hepatitis C tests are negative. A decision is made to refer him to the emergency department (ED) for admission and surgical intervention.

**1.1 Compare the definitions of opiate and opioid.**

**1.2 Match the receptor types that opioids bind to with their corresponding endogenous ligands. (Each answer choice can be used once.)**

| Receptor Type | Ligand |
| --- | --- |
| ___ 1. Mu (MOP) | a. Dynorhins |
| ___ 2. Delta (DOP) | b. Enkephalins |
| ___ 3. Kappa (KOP) | c. Endorphins |
|  |  |

**1.3 Describe the mechanism of full agonist (e.g. heroin/morphine or fentanyl) at the mu opioid receptor.**

**1.4 List the resultant physiologic effects, including adverse effects, that are caused by full agonist ligand activation of the mu opioid receptor.**

**1.5 Describe the organ system/location of receptors primarily causing these physiologic effects (for example: analgesia – central nervous system)**

**1.6** **Which property contributes to the heightened ability of fentanyl to produce opioid toxicity as compared to heroin/morphine? Select all correct answers.**

1. *Fentanyl is about 50-100 x more potent than morphine.*
2. *Fentanyl is about 50 – 100 x less potent than morphine.*
3. *Fentanyl is more lipophilic-crosses the blood brain barrier faster than morphine.*
4. *Fentanyl is less lipophilic-crosses the blood brain barrier slower than morphine.*
5. *Fentanyl is short acting, so you need to dose more often to maintain an effect.*
6. *Fentanyl is long acting, so you need to dose less often to maintain an effect.*

**1.7 Compare pharmacokinetics of different full agonists (fentanyl, heroin, methadone) by matching the full agonist with the corresponding half-life**

| *Full agonist* | *Half Life* |
| --- | --- |
| *___1. Fentanyl* | *a. 2-4 hours* |
| *___2. Morphine* | *b. 12- 24 hours* |
| *___3. Methadone* | *c. 30 minutes* |

**1.8 The route of administration affects pharmacokinetics and pharmacodynamics of various opioids (fentanyl, morphine, heroin, codeine, methadone). Modes of delivery include oral/first pass, IV, lozenges/oral mucosa, nasal, local/epidural, subcutaneous, transdermal (patch), intrathecal, intramuscular.**

a) How is fentanyl metabolized (which organ, which enzyme)? Is there more bioavailable fentanyl via oral delivery compared to intravenous or transdermal (skin patch) delivery of fentanyl?

b) Is there more bioavailable morphine available via oral delivery compared to intravenous? What is morphine metabolized into, and by what enzymes?

c) How is heroin metabolized into morphine?

d) How is codeine metabolized into morphine?

e) How is methadone metabolized? Does methadone have higher bioavailability than morphine?

**1.9 Describe drug interactions (hint: consider mechanism of drug metabolism) and pharmacogenetics that are important in oral delivery of opioids.**

**Section 2**

At the emergency department, the patient informs the physicians that he is having severe arm pain. The physicians refuse to give the patient opioids because of his history of opioid use disorder. The nurse administers and obtains a high score on the Clinical Opioid Withdrawal Scale (COWS), but the physicians do not want to exacerbate his addiction by giving him opioids. The patient is distraught, and he walks down the hall to use the restroom.

**2.1 Which signs and symptoms of opioid withdrawal are measured by COWS?**

- 1. **With repeated use of opioids, patients become tolerant to the effect of opioids (analgesia, etc.). However, what are two effects of opioids that patients DO NOT become tolerant to? How does down-regulation of receptors relate to tolerance? What is opioid-induced hyperalgesia?**

**Section 3:**

The patient comes out of the bathroom and returns to his stretcher in the ED. Within a few minutes, the patient becomes unresponsive, with shallow breaths and a respiratory rate of 6. Oxygen saturation is 86% on room air. The bedside nurse notes that his pupils are pinpoint and alerts the ED physician.

**3.1 What are signs and symptoms of opioid overdose? (Note that the patient described in this clinical vignette does not exhibit all of the signs and symptoms of opioid overdose. Please list signs/symptoms that this patient exhibits, in addition to other signs/symptoms associated with opioid overdose.)**

The emergency medicine physician administers intranasal naloxone

**3.2 Discuss the pharmacodynamic properties of naloxone**

**3.3 Discuss the pharmacokinetics i.e., half-life, of naloxone in comparison to injected opioids, and why there is a need to monitor patients for respiratory depression after administering the first dose of naloxone.**

**3.4 Describe the different formulations of naloxone (oral, intranasal, intravenous, intramuscular), including pharmacokinetics.**

| *Naloxone Formulations:* | *Bioavailability:* |
| --- | --- |
| *Oral* |  |
| *Intranasal spray* |  |
| *Intravenous (IV)* |  |
| *Intramuscular (IM)* |  |

After receiving 1 mg of intramuscular naloxone, the patient becomes more alert and his respiratory rate increases to normal. He is placed on a pulse oximetry monitor to keep track of his oxygen saturation. He appears restless. He then starts to vomit and grabs his abdomen, reporting cramping abdominal pains and muscle aches. He reports having used heroin in the restroom because his arm was in such severe pain and he was starting to feel “dope sick”.

**Section 4. Treatment for Substance Use Disorder**

His labs in the ED show white blood cell count of 22,000 with 90% neutrophils (elevated white blood cell counts are often seen in infections). Ultrasound confirms 3cm x 3cm x 3cm abscess in his right forearm. After 8 hours in the ED, and initiation of broad-spectrum antibiotics, he is admitted to the medical floor and the orthopedic hand surgery service is consulted. They recommend that he undergo an incision and drainage procedure to treat the abscess. A decision is made to treat Mr. G’s pain with intravenous hydromorphone peri-operatively.

He undergoes a successful incision and drainage procedure by the orthopedic hand service. The patient’s nurse calls the medical team on post-operative day 1 and states, “Your intravenous drug abuser keeps asking for pain meds. He never stops complaining.” The intern checks the computer to see that Mr. G is receiving his hydromorphone every four hours as prescribed. On post-operative day 3, the orthopedic hand service states that Mr. G will not have to return to the OR for any further procedures. The patient states that he is ready to treat his addiction, and he asks if he can be started on medications to treat opioid use disorder. His pain in his right upper extremity has improved.

**4.1 Name the three FDA-approved medications for opioid use disorder and explain their pharmacodynamics and pharmacokinetics properties that are important as for their efficacy as treatments.**

| **Drug Treatment** | **Pharmacodynamics (mu receptors)** | **Half-Life** | **Oral Bioavailability (Good vs. Poor)** | **Primary Route(s) of Delivery (oral, IV, sublingual, transdermal, etc., and there may be more than one correct answer)** | **Other properties (will be discussed by facilitator)** |
| --- | --- | --- | --- | --- | --- |
| **Methadone** |  |  |  |  |  |
| **Buprenorphine** |  |  |  |  |  |
| **Naltrexone** |  |  |  |  |  |

- 1. **Reflect on the nurse’s word choice with regards to professionalism**

Optional Reference: Botticelli MP, Koh HK. Changing the Language of Addiction. *JAMA*. 2016;316(13):1361-1362. doi:10.1001/jama.2016.11874

Wrap-up

Mr. G is accepted into a three-month inpatient substance use rehabilitation program and thanks the team for their attentiveness to his care. Mr. G feels optimistic for his future on the day of discharge.
