# Supplementary material for: Opioid Use Disorder Curriculum: Preclerkship Pharmacology Case-Based Learning Session
Source: MedEdPORTAL. 2022 May 10;18:11255. doi: 10.15766/mep_2374-8265.11255 (PMC9085981; doi:10.15766/mep_2374-8265.11255)
Supplement: Supplementary file 1 — Case Instructions and Resources.docxCase - Student Version.docxCase - Facilitator Guide.docxCase - Figures.pptPharmacology Exam Questions.docxEvaluation Questions.docx [file mep_2374-8265.11255-s001.zip › C. Case - Facilitator Guide.docx]

**Medications for Opioid Use Disorder: Pharmacology Case-Based Learning Session — Facilitator Guide**

1. **Educational Objectives:**

By the end of this activity, learners will be able to:

• Describe the physiologic effects and pharmacology of opioids

• Apply the pharmacology of opioids to the medications used to treat opioid use disorder

• Explain how pharmacology fundamentals are important in the real world of clinical medicine

This CBL is based on an opioid clinical case. It was created by pharmacology faculty in collaboration with clinical faculty who have experience in treating patients with opioid use disorder. The goal of the session is to reinforce pharmacology fundamentals students already learned, and to help students learn how these fundamentals apply in the real world of clinical medicine. Students will already have had lectures on the following topics and tested on part of it:

Pharmacodynamics, Pharmacokinetics, Drug Metabolism, Pharmacogenetics/genomics

G coupled receptors, Autonomics

Some lectures in Physiology are relevant to this case.

To prepare for the session, students were recommended to review the material that they already learned in class AND to do research on their own to answer the questions/topics in the CBL study guide provided. We expect students to come prepared for the in-class session to discuss the clinical case and underlying pharmacology. Students were also recommended to review the following optional resources in advance of this CBL session to help them prepare.

Recommended videos on opioids and opioid use disorder:

Khan Academy. Treatments and triggers for drug dependence. <https://youtu.be/tPhcRBkVmUM> June 25, 2014. Accessed September 24, 2021.

Medicurio. Opioid Drugs, Part 1. Mechanism of Action. <https://youtu.be/s60KzN4GJdQ> June 28, 2018. Accessed September 24, 2021.

DRUGBANK

Wishart DS, Feunang YD, Guo AC, et al. DrugBank 5.0: a major update to the DrugBank database for 2018. *Nucleic Acids Res*. 2018;46(D1):D1074-D1082. doi:10.1093/nar/gkx1037

PubChem:

Kim S, Chen J, Cheng T, et al. PubChem in 2021: new data content and improved web interfaces. *Nucleic Acids Res*. 2021;49(D1):D1388–D1395. [doi:10.1093/nar/gkaa971](https://doi.org/10.1093/nar/gkaa971)

Katzung BG, Vanderah TW. Katzung B.G., & Vanderah T.W.(Eds.),Eds. Bertram G. Katzung, and Todd W. Vanderah.eds. *Basic & Clinical Pharmacology, 15e*. McGraw Hill; 2021.

**B. Room assignments:** There are 8 groups for this CBL exercise. There are two faculty leaders for each group, a basic science and a clinical faculty member.

**C. Other**

- Students were instructed to attend the small group session to which they were assigned, an to please be prompt.
- The faculty leaders will expect all students to participate. Students will provide their name when answering a question, so that facilitators can note it on their rosters.

ATTENTION, STUDENTS: If you are accessing this material BEFORE it is used in your course, please do NOT read this document prior to the class session. An answer key is included in this module, which is designed to lead you through a learning experience that reinforces your knowledge of the content. Early review or dissemination of this material to others will diminish the learning opportunity and be considered academic misconduct.

***Time check: 0 minutes.***

***Total amount of time allowed for the case is 60 minutes.***

**Section 1:**

Symptom: Upper extremity pain and swelling.

Mr. G is a 40-year-old man who presents to the Syringe Services Program with a chief complaint of a 3-day history of worsening right upper extremity pain and swelling. His medical history is significant for opioid use disorder, and he reports recent syringe sharing and re-use.

On physical exam, the patient is afebrile. The distal right upper extremity is swollen, warm, erythematous, and tender to light palpation. Point of care ultrasound shows an abscess on the ventromedial/flexor surface of his forearm. Rapid HIV and hepatitis C tests are negative. A decision is made to refer him to the emergency department (ED) for admission and surgical intervention.

**1.1 Compare the definitions of opiate and opioid.**

*Answer:*

*Opiate: natural (e.g. opium from poppy plant)*

*Opioid: all compounds that work at opioid receptors, mainly synthetic*

**1.2 Match the receptor types that opioids bind to with their corresponding endogenous ligands. (Each answer choice can be used once.)**

| Receptor Type | Ligand |
| --- | --- |
| ___ 1. Mu (MOP) | a. Dynorhins |
| ___ 2. Delta (DOP) | b. Enkephalins |
| ___ 3. Kappa (KOP) | c. Endorphins |
|  |  |
| *Answer:* |  |

*__c._ 1. Mu (MOP) Answer: c. Endorphins*

*__b._ 2. Delta (DOP) Answer: b. Enkephalins*

*__a._ 3. Kappa (KOP) Answer: a. Dynorphins*

*Note: Don’t confuse “mu” with muscarinic M1-M5*

*(Note: some students may bring up nociceptin/orphanin FQ peptide receptor (NOP). While both the NOP receptor and its endogenous ligand N/OFQ have structural and functional similarity to the other three opioid receptors and their endogenous ligands respectively, the NOP receptor does not bind to classical opioid ligands, and neither does the endogenous NOP ligand N/OFQ bind to the other opioid receptors, making the NOP–N/OFQ receptor-ligand system a class in itself, distinct from the opioid family in several important ways.)*

**1.3 Describe the mechanism of full agonist (e.g. heroin/morphine or fentanyl) at the mu opioid receptor.**

*Answer:*

- *Mechanism of Signaling: All opioid receptors are GPCRs and signal through Gi to reduce adenylate cyclase activity intracellularly. (See slide 2)*
- *Reduces presynaptic calcium conductance, decreases excitatory neurotransmitter release. (See slide 3)*
- *Opens post synaptic K+ channels and hyperpolarizes, which inhibits post synaptic neurons. Overall depresses activity of neural transmission at targets (reduced neuronal excitability). (See slide 3)*

**1.4 List the resultant physiologic effects, including adverse effects, that are caused by full agonist ligand activation of the mu opioid receptor.**

*Answer:*

*Physiological effects: analgesia, euphoria, respiratory depression, constipation, pruritus, miosis and immunosuppression*

**1.5 Describe the organ system/location of receptors primarily causing these effects (for example: analgesia – central nervous system)**

*Answer:*

- *Analgesia: Brain/CNS*
- *Euphoria: Brain/CNS*
- *Respiratory depression: Respiratory center in nervous system (brain stem/mid brain)*
- *Constipation: local in the gut*
- *Pruritus: stimulates histamine release from mast cells*
- *Miosis: Opioid receptors are present on sympathetic nerves of the iris, and the pupillary effect has been largely attributed to changes within the oculomotor center*
- *Immune suppression-immune cells. Immune cells express all three opioid receptors.*

*Clinical pearl: since immune cells express all three opioid receptors, opioid use increases susceptibility to opportunistic infection*

***Time check: 12 minutes (48 minutes remaining)***

**1.6 Which property contributes to the heightened ability of fentanyl to produce opioid toxicity as compared to heroin/morphine? Select all correct answers.**

1. *Fentanyl is about 50-100 x more potent than morphine.*
2. *Fentanyl is about 50 – 100 x less potent than morphine.*
3. *Fentanyl is more lipophilic-crosses the blood brain barrier faster than morphine.*
4. *Fentanyl is less lipophilic-crosses the blood brain barrier slower than morphine.*
5. *Fentanyl is short acting, so you need to dose more often to maintain an effect.*
6. *Fentanyl is long acting, so you need to dose less often to maintain an effect.*

*Answer:*

*a. Fentanyl is about 50-100 x more potent than morphine. (See slide 4). Fentanyl has a higher risk of causing overdose because a low dose is more likely to produce respiratory depression.*

*c. Fentanyl is more lipophilic-crosses the blood brain barrier faster than morphine.*

*e. Fentanyl is short acting, so you need to dose more often to maintain an effect. (The half-life is addressed in the following question).*

*(Note: methadone is also a full agonist, similar to morphine)*

*Clinical pearls: Increased frequency of injection increases risk of transmitting infectious diseases. When fentanyl infiltrated the heroin supply, people who inject drugs (PWID) injected more frequently, leading to outbreaks of HIV and Hepatitis C. Fentanyl also caused a surge in the overdose rates in the US because of increased potency.*

**1.7 Compare pharmacokinetics of different full agonists (fentanyl, heroin, methadone) by matching the full agonist with the corresponding half-life**

| *Full agonist* | *Half Life* |
| --- | --- |
| *___1. Fentanyl* | *a. 2-4 hours* |
| *___2. Morphine* | *b. 12- 24 hours* |
| *___3. Methadone* | *c. 30 minutes* |

*Answer:*

*Half lives in humans:*

1. *Fentanyl : c. 30 min (clinical pearl: used in anesthesia since removed rapidly)*
2. *Morphine: a. 2-4 hours (clinical pearl: better for postoperative pain)*
3. *Methadone: b. 12-24 hrs (clinical pearl: better for postoperative pain)*

**1.8 The route of administration affects pharmacokinetics and pharmacodynamics of various opioids (fentanyl, morphine, heroin, codeine, methadone). Modes of delivery include oral/first pass, IV, lozenges/oral mucosa, nasal, local/epidural, subcutaneous, transdermal (patch), intrathecal, intramuscular.**

a) How is fentanyl metabolized (which organ, which enzyme)? Is there more bioavailable fentanyl via oral delivery compared to intravenous or transdermal (skin patch) delivery of fentanyl?

*Answer: Fentanyl is metabolized in the liver (and small intestine) by CYP3A4, and very little active parent compound is bioavailable via* ***oral delivery.*** *Therefore, the most common delivery is* ***IV or transdermal*** *(skin patch). It is important to note that touching fentanyl will not cause overdose.*

b) Is there more bioavailable morphine available via oral delivery compared to intravenous? What is morphine metabolized into, and by what enzymes?

*Answer: Morphine has low oral bioavailability. Need 6x higher oral morphine dose than parenteral (through the IV) in order to achieve the same physiologic effect. Also, morphine (and other opioids with free hydroxyl groups) are conjugated by phase 2 enzymes to morphine 3-glucuronide (M3G) or morpine-6-glucoronide (M6G). Also metabolized in other tissues by these enzymes, which limits bioavailabilty to ~35%. M3G has neuroexcitatory effects and can cause seizures, but probably not through mu opioid receptors. 10% of morphine is conjugated to M6G, which has 4-6 x analgesic potency of morphine. But it does not cross the blood brain barrier well.*

c) How is heroin metabolized into morphine?

*Answer: Heroin is diacetyl morphine. Hydrolyzed by tissue and plasma esterases to monoacetylmorphine and then morphine. (Poor oral availability.)*

d) How is codeine metabolized into morphine?

*Answer: Codeine is a prodrug, metabolized (demethylated) to morphine in the liver by CYP2D6, then glucuronidated. Similar for the others (oxycodone to oxymorphone to O3G; hydrocodone to hydromorphone to H3G). Thus, oral delivery is important for these drugs to work. (Oxycodone also metabolized by CYP3A4).*

e) How is methadone metabolized? Does methadone have higher bioavailability than morphine?

*Answer: Methadone: mostly metabolized by CYP3A4 and CYP2B6 (other cytochrome P450’s contribute).*

*Hydroxyl Conjugates excreted in the kidney. No hydroxyl (OH) so not metabolized by glucuronidases. Overall higher bioavailability than morphine.*

***Time check: 22 minutes (38 minutes remaining)***

**1.9 Describe drug interactions (hint: consider mechanism of drug metabolism) and pharmacogenetics that are important in oral delivery of opioids:**

*Answer:*

*Drug Interactions:*

*First pass effect of liver metabolism*

*CYP3A4 metabolizes 25-50% of all drugs so there can be high risk of drug interactions. If CYP3A4 is inhibited (by agents such as grapefruit juice, antiretrovirals), this can cause adverse effects of opioids because the opioids will not be metabolized as efficiently for excretion (leading to increased analgesia, respiratory depression). CYP3A4 inducers can reduce analgesic efficacy. Examples of CYP3A4 inducers include cafestol in unfiltered coffee, anticonvulsants****,*** *statins.*

*CYP2D6 is involved in the metabolism of opioids such as tramadol, codeine, hydrocodone, and oxycodone. Inhibitors of CYP2D6 include SSRI’s (e.g.,citalopram), ritonavir, bupropion, methadone, and terbinafine. CYP2D6 inducers include corticosteroids. CYP2D6 is not very susceptible to enzyme induction. Thus, pharmacogenetics, rather than drug therapy, accounts for most ultrarapid CYP2D6 metabolizers.*

***Pharmacogenetics:***

*Pharmacogenetics are a major factor for CYP2D6:*

*5% to 10% of Caucasians possess low metabolizer variants of the CYP2D6 gene leading to reduced clearance of drugs metabolized by this isoenzyme. Variable in African populations (estimated up to 34%).*

*1% -7% of Caucasians have CYP2D6 variants associated with rapid metabolism. Prevalence of rapid metabolizers in African populations is from 9%- 30%. This can cause either lower analgesic effects for some opioids, but for drugs like codeine and tramadol, this can cause toxicity because codeine will be over-converted to morphine.*

*Clinical pearl: People who inject drugs have high rates of HIV, complications from Staphylococcus infections like osteomyelitis, and tuberculosis. A common treatment for some of these diseases includes rifampin, which can increase analgesic needs due to drug interactions (rifampin is an inducer of CYP3A).*

*Clinical pearl: Methadone can also increase the risk for cardiac arrhythmias. Methadone cause QT prolongation through direct effects on the resting membrane potential and represent a risk of medication interactions with other drugs that can cause QT prolongation, such as certain second generation antipsychotics.*

*Important factors in pain management.*

*-Liver dysfunction (e.g. caused by hepatitis C or alcohol use disorder) can affect metabolism of oral opioids.*

*Clinical pearl: the more frequently a person injects drugs, this increases the person’s risk of acquiring infectious diseases like hepatitis C, which can be transmitted through sharing of needles.****Time check: 30 minutes (30 minutes remaining)***

**Section 2**

At the emergency department, the patient informs the physicians that he is having severe arm pain. The physicians refuse to give the patient opioids because of his history of opioid use disorder. The nurse administers and obtains a high score on the Clinical Opioid Withdrawal Scale (COWS), but the physicians do not want to exacerbate his addiction by giving him opioids. The patient is distraught, and he walks down the hall to use the restroom.

**2.1 Which signs and symptoms of opioid withdrawal are measured by COWS?**

*Answer:*

- *COWS can be used to determine the severity of opioid withdrawal (See slide 5)*
- *Included signs and symptoms in COWS are: Tachycardia, diaphoresis, restlessness, pupillary dilation, bone or joint aches, rhinorrhea or lacrimation, diarrhea, tremor, yawning, anxiety or irritability, gooseflesh skin*
  1. **With repeated use of opioids, patients become tolerant to the effect of opioids (analgesia, etc.). However, what are two effects of opioids that patients DO NOT become tolerant to? How does down-regulation of receptors relate to tolerance? What is opioid-induced hyperalgesia?**

*Answer:*

- *Patients become tolerant to all of the effects of opioids, with the exception of constipation and miosis. With repeated opioid use, it is theorized that there is a down-regulation of the receptors, which leads to decreased physiologic response to the same dose of opioids. This compensation is due to overstimulation of opioid receptor.*
- *Opioid-induced hyperalgesia is a state of nociceptive sensitization cause by exposure to opioids (mechanism is not well understood)*
- *Clinical pearl: due to tolerance, patients with OUD often require higher doses of opioid analgesics than patients without OUD in order to achieve the same physiologic effects*

***Time check: 34 minutes (26 minutes remaining)***

**Section 3:**

The patient comes out of the bathroom and returns to his stretcher in the ED. Within a few minutes, the patient becomes unresponsive, with shallow breaths and a respiratory rate of 6. Oxygen saturation is 86% on room air. The bedside nurse notes that his pupils are pinpoint and alerts the ED physician.

**3.1 What are signs and symptoms of opioid overdose? (Note that the patient described in this clinical vignette does not exhibit all of the signs and symptoms of opioid overdose. Please list signs/symptoms that this patient exhibits, in addition to other signs/symptoms associated with opioid overdose.)**

*Answer: Depressed mental status, decreased respiratory rate, decreased tidal volume, cyanosis, decreased bowel sounds, miotic pupils.*

*-Clinical pearl: Respiratory rate < 12 is the best predictor of overdose*

The emergency medicine physician administers intranasal naloxone

**3.2 Discuss the pharmacodynamic properties of naloxone**

Answer:

*Naloxone (Narcan) is a high-affinity competitive opioid* ***antagonist****. Naloxone will displace the heroin/morphine or fentanyl and shift the dose response curve to the right. Upon opioid overdose, use of naloxone will rapidly reverse the respiratory depression. Naloxone can cause rapid withdrawal. (See slide 6)*

**3.3 Discuss the pharmacokinetics i.e., half-life, of naloxone in comparison to injected opioids, and why there is a need to monitor patients for respiratory depression after administering the first dose of naloxone.**

*Answer:*

*Naloxone’s effects last for 30-60 min. This is a rapid half-life compared to morphine (3-4 hours), but more similar to fentanyl. Multiple doses of naloxone may be needed because the original injected drug may continue to be present in the system and have continued respiratory depression effects after naloxone is cleared. Must monitor patient to be sure they do not show further respiratory depression.*

- 1. **Describe the different formulations of naloxone (oral, intranasal, intravenous, intramuscular), including pharmacokinetics.**

| *Naloxone Formulations:* | *Bioavailability:* |
| --- | --- |
| *Oral* |  |
| *Intranasal spray* |  |
| *Intravenous (IV)* |  |
| *Intramuscular (IM)* |  |

*Answer:*

| *Naloxone Formulations:* | *Bioavailability:* | *Other comments:* |
| --- | --- | --- |
| *Oral* | *Bioavailability is poor <2%; would need very high oral doses to antagonize systemic opioid overdose* | *First pass metabolism to naloxone-3-glucuronide, excreted* |
| *Intranasal spray* | *Good bioavailability; can observe significant plasma naloxone within 2-5 minutes* | *0.1 ml spray delivers 4mg dose of naloxone; peak similar to IM* |
| *Intravenous (IV)* | *Good bioavailability; distributes in about 1-2 minutes* | *If no effect after naloxone 10 mg IV, then patient may not be in opioid induced toxicity* |
| *Intramuscular (IM)* | *Good bioavailability 2-5 minutes with peak by 10-15 minutes* | *Similar to intranasal spray* |

After receiving 1mg intramuscular naloxone , the patient becomes more alert and his respiratory rate increases to normal. He is placed on a pulse oximetry monitor to keep track of his oxygen saturation. He appears restless. He then starts to vomit and grabs his abdomen, reporting cramping abdominal pains and muscle aches. He reports having used heroin in the restroom because his arm was in such severe pain and he was starting to feel “dope sick.”

***Time check: 45 min (15 minutes remaining)***

**Section 4. Treatment for Substance Use Disorder**

His labs in the ED show white blood cell count of 22,000 with 90% neutrophils. Ultrasound confirms 3cm x 3cm x 3cm abscess in his right forearm. After 8 hours in the ED, and initiation of broad-spectrum antibiotics, he is admitted to the medical floor. The orthopedic hand surgery service is consulted; they recommend that he undergo an incision and drainage procedure to treat the abscess. A decision is made to treat Mr. G’s pain with intravenous hydromorphone peri-operatively.

He undergoes a successful incision and drainage procedure by the orthopedic hand service. The patient’s nurse calls the medical team on post-operative day 1 and states, “Your intravenous drug abuser keeps asking for pain meds. He never stops complaining.” The intern checks the computer to see that Mr. G is receiving his hydromorphone every four hours as prescribed. On post-operative day 3, the orthopedic hand service states that Mr. G will not have to return to the operating room for any further procedures. The patient states that he is ready to treat his addiction, and he asks if he can be started on medications to treat opioid use disorder. His pain in his right upper extremity has improved.

**4.1 Name the three FDA-approved medications for opioid use disorder and explain their pharmacodynamics and pharmacokinetics properties that are important as for their efficacy as treatments.**

|  |  |  |  |  |  |
| --- | --- | --- | --- | --- | --- |
|  |  |  |  |  |  |
|  |  |  |  |  |  |
|  |  |  |  |  |  |

Refer to figures on slides 7 and 8, and to table on slide 9. See below for the completed table


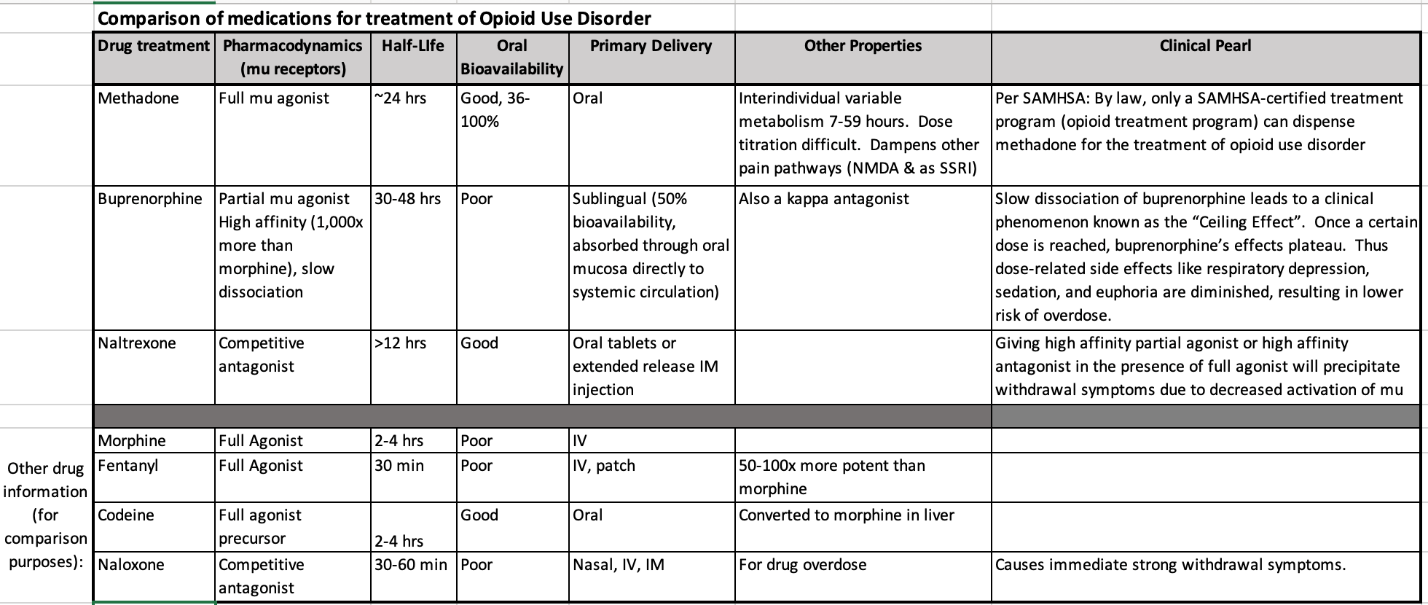


*Clinical Pearl:* Suboxone (buprenorphine - naloxone) is a sublingual medication. Naloxone (an opioid antagonist) is included in Suboxone to prevent inappropriate IV use of the buprenorphine component.

**4.2 Reflect on the nurse’s word choice with regards to professionalism**

Optional Reference: Botticelli MP, Koh HK. Changing the Language of Addiction. *JAMA*. 2016;316(13):1361-1362. doi:10.1001/jama.2016.11874

- *Nurse was unprofessional in that she used language that is stigmatizing.*
- *Negative attitudes by professionals have been found to adversely affect quality of care and treatment outcomes.*
- *There are better word choice options, referred to as “Person-first” language (clinical, non-stigmatizing language to replace negative terms that have been used to label people).*
- *Example: “Person with a substance use disorder” should be used instead of “Abuse/abuser/addict/alcoholic” which are words that negatively affect judgements about people with substance use disorder.*
- *Example: Use words like: “Person in recovery” or “not currently using substances.” Avoid: “clean” and “dirty.”*
- *Example: Use words like “Medication assisted treatment” instead of “replacement” or “substitute.”*

*The document that is hyperlinked to the article contains further details.*

Wrap-up

Mr. G is accepted into a three-month inpatient substance use rehabilitation program and thanks the team for their attentiveness to his care. Mr. G feels optimistic for his future on the day of discharge.

***Time check: 60 minutes (0 minutes remaining)***
