# Supplementary material for: Opioid Use Disorder Curriculum: Preclerkship Pharmacology Case-Based Learning Session
Source: MedEdPORTAL. 2022 May 10;18:11255. doi: 10.15766/mep_2374-8265.11255 (PMC9085981; doi:10.15766/mep_2374-8265.11255)
Supplement: Supplementary file 1 — Case Instructions and Resources.docxCase - Student Version.docxCase - Facilitator Guide.docxCase - Figures.pptPharmacology Exam Questions.docxEvaluation Questions.docx [file mep_2374-8265.11255-s001.zip › E. Pharmacology Exam Questions.docx]

**Pharmacology Exam Questions**

Question 1:

NBME Customized Assessment question

Patient vignette identified using keyword “opioid”

Question 2:

NBME Customized Assessment question

Patient vignette identified using keyword “opioid”

Question 3:

Written by Sandra Lemmon, PharmD

Which of the following characteristics of Buprenorphine make it a useful treatment for opioid use disorder:

A. It is a competitive antagonist for mu-opioid receptors with a long half-life, so it precipitates withdrawal, but its longer effects give users more chance to adapt to abstinence from opioid abuse.

B. It is a high affinity full agonist for mu-opioid receptors with a longer half-life (>24 hours), so the need for drug use is much less frequent.

C. It is a partial agonist for the mu-opioid receptor, so its effects are less extreme, diminishing euphoria and risks of respiratory depression.

D. It is a full agonist with a short half-life, so its effects don’t last too long, and there is less risk of overdose.

E. When taken orally (swallowed) it has good bioavailability, so it is easy for patients to take.

Correct Answer: C

Explanation:

Educational objective: Explain how pharmacology fundamentals are important in the real world of clinical medicine.

Correct Answer: C

Patients with opioid use disorder often use opioids such as heroin and prescription pain medications (e.g., fentanyl); these drugs are full agonists that stimulate µ receptors. Full agonist ligand activation of the µ opioid receptor results in physiologic effects such as analgesia and respiratory depression (decreased respiratory rate).

Buprenorphine is an effective treatment for opioid use disorder because it is a partial agonist at the µ receptors. Therefore, the µ receptors are less activated, so buprenorphine’s effects are less extreme as compared to a full agonist, diminishing euphoria and risks of respiratory depression. Buprenorphine has a higher affinity for the µ receptors compared to morphine. The slow dissociation of buprenorphine leads to a clinical phenomenon known as the “ceiling effect.” Once a certain dose is reached, buprenorphine’s effects plateau. Thus, dose-related side effects like respiratory depression, euphoria, and sedation are diminished, resulting in lower risk of overdose.

Extra clinical pearls: buprenorphine is an antagonist to kappa-opioid receptors, while naloxone and naltrexone are not. Thus, kappa-induced dysphoria is unopposed by naloxone and naltrexone, while being counter-acted by buprenorphine. As a result, patients on buprenorphine usually have euthymia rather than the dysphoria they would display under mu-antagonists.

Option A is incorrect because buprenorphine is a partial agonist for the µ opioid receptor. (However, it is correct that buprenorphine has a long half-life (30-48 hours) and can precipitate withdrawal symptoms.)

Option B is incorrect because buprenorphine is a partial agonist. It is correct that buprenorphine has a higher affinity for the µ opioid receptor than morphine, thereby blocking the effect of other drugs (such as fentanyl, which is broken down to morphine in the body).

Option D is incorrect because buprenorphine is a partial agonist with a long half-life, and a “ceiling effect” (described above).

Option E is incorrect because buprenorphine has poor bioavailability. Buprenorphine’s bioavailability is higher when administered sublingually (50% bioavailability, absorbed through oral mucosa directly into the systemic circulation).

Note: question could be re-written as:

A 38-year-old woman with a history of opioid use disorder presents to her primary care physician. She states that she would like to stop injecting heroin and she is interested in starting medications to help her. Her physician recommends buprenorphine. Which of the following explains why buprenorphine is effective in the treatment of opioid use disorder?
