# Supplementary material for: Opioid Use Disorder Curriculum: Preclerkship Pharmacology Case-Based Learning Session
Source: MedEdPORTAL. 2022 May 10;18:11255. doi: 10.15766/mep_2374-8265.11255 (PMC9085981; doi:10.15766/mep_2374-8265.11255)
Supplement: Supplementary file 1 — Case Instructions and Resources.docxCase - Student Version.docxCase - Facilitator Guide.docxCase - Figures.pptPharmacology Exam Questions.docxEvaluation Questions.docx [file mep_2374-8265.11255-s001.zip › F. Evaluation Questions.docx]

**Evaluation Questions**

| Student Evaluations of CBL | Strongly Agree | Agree | Disagree | Strongly Disagree | Not Applicable |
| --- | --- | --- | --- | --- | --- |
| The Case Based Learning session (on opioids) is an effective way to learn pharmacology principals (check one) |  |  |  |  |  |
| The Case Based Learning session (on opioids) reinforced pharmacological fundamentals learned (check one) |  |  |  |  |  |
| The Case Based Learning session (on opioids) showed how pharmacology fundamentals are important in the real world of clinical medicine (check one) |  |  |  |  |  |
